# Contributions of lower extremity kinematics to trunk accelerations during moderate treadmill running

**DOI:** 10.1186/1743-0003-11-162

**Published:** 2014-12-12

**Authors:** Timothy R Lindsay, James A Yaggie, Stephen J McGregor

**Affiliations:** School of Health Promotion & Human Performance, Eastern Michigan University, Ypsilanti, MI USA; School of Health Sciences & Human Performance, Ithaca College, Ithaca, NY USA

**Keywords:** High resolution accelerometers, Root mean square, Principal components analysis, Running, Economy, Injury, Stiffness

## Abstract

**Background:**

Trunk accelerations during running provide useful information about movement economy and injury risk. However, there is a lack of data regarding the key biomechanical contributors to these accelerations. The purpose was to establish the biomechanical variables associated with root mean square (RMS) accelerations of the trunk.

**Methods:**

Eighteen healthy males (24.0 ± 4.2 yr; 1.78 ± 0.07 m; 79.7 ± 14.8 kg) performed treadmill running with high resolution accelerometer measurement at the lumbar spine and full-body optical motion capture. We collected 60 sec of data at three speeds (2.22, 2.78, 3.33 m∙s^−1^). RMS was calculated for medio-lateral (ML), anterio-posterior (AP), vertical (VT), and the resultant Euclidean scalar (RES) acceleration. From motion capture, we calculated 14 kinematic variables, including mean sagittal plane joint angles at foot contact, mid-stance, and toe-off. Principal components analysis (PCA) was used to form independent components comprised of combinations of the original variables. Stepwise regressions were performed on the original variables and the components to determine contributions to RMS acceleration in each axis.

**Results:**

Significant speed effects were found for RMS-accelerations in all axes (p < 0.05). Regressions of the original variables indicated from 4 to 5 variables associated with accelerations in each axis (*R*^2^ = 0.71 to 0.82, p < 0.001). The most prominent contributing variables were associated with the late flight and early stance phase. PCA reduced the data into four components. Component 1 included all hip angles before mid-stance and component 2 was primarily associated with propulsion. Regressions indicated key contributions from components 1 and 2 to ML, VT, and RES acceleration (p < 0.05).

**Conclusions:**

The variables with highest contribution were prior to mid-stance and mechanically relate to shock absorption and attenuation of peak forces. Trunk acceleration magnitude is associated with global running variables, ranging from energy expenditure to forces lending to the mechanics of injury. These data begin to delineate running gait events and offer relationships of running mechanics to those structures more proximal in the kinetic chain. These relationships may provide insight for technique modification to maximize running economy or prevent injury.

**Electronic supplementary material:**

The online version of this article (doi:10.1186/1743-0003-11-162) contains supplementary material, which is available to authorized users.

## Background

Running is an increasingly popular sport that provides substantial health benefits at minimal expense. Estimates from 2011 are that 38.7 million Americans participate in running or jogging 6 or more days per year (up from 24.5 million in 2001), with 9.2 million doing so 110 or more days per year (up from 6.8 million in 2001) [1]. Offsetting the numerous health benefits of exercise is the relatively high incidence of injury, which according to one systematic review, ranges from 19-79% [2]. Even at the lower end of this range, the high participation rate means that injury is a substantial concern. Since most running injuries are chronic rather than acute [3, 4], the tolerable level of accumulated stress is an important consideration. This stress depends on multiple factors including the training dose, anatomical structure, and movement mechanics [4–7]. We focus on mechanics in this paper.

Mechanically speaking, running involves the application of force to the ground to generate the resultant ground reaction force (GRF) necessary for forward propulsion and support against gravity. This places stress on soft tissue and bone via force transmission through the kinetic chain, which may lead to future injury if the exercise dosage exceeds regenerative capacity. A comprehensive description of forces requires a complicated model, but the acceleration of the center of mass (COM) can provide a simple quantification of net force. Continuous COM data may then be expressed as a root mean square (RMS) value to represent the overall magnitude of acceleration over many strides [8]. RMS provides a measure of dispersion similar to standard deviation, only relative to zero rather than the mean [9]. The presence of more extreme values in the signal (i.e., high acceleration or deceleration) increases the RMS value. Acceleration at any anatomical location depends on the level of attenuation through tissue deformation and joint excursion at all points distal. The attenuation of force and acceleration can be modified with lower limb stiffness and may alter the likelihood of running-related injuries [10, 11]. High stiffness may aid performance and economy but also may increase the risk of injury to structural components. In contrast, stiffness that is too low may be metabolically costly and increase the risk of soft tissue injury [8, 10–12]. Stiffness depends on the intrinsic properties of bone and soft tissue (muscle, tendon, ligament, and cartilage) [13], but also may be modified via kinematic changes. For example, in subjects instructed to perform a soft drop landing, there was greater knee joint excursion [14, 15]. As well, Derrick [11] has argued that runners generally run with extended knees prior to impact, but are able to increase knee flexion in order to reduce vertical accelerations. Similarly, subjects who were instructed to adopt a “Groucho running” style had longer strides (believed to be associated with decreased stiffness) and decreased stiffness, as directly measured [16]. Interventions such as gait retraining to pursue this objective are promising and demonstrate that kinematics are modifiable [17, 18].

There has not yet been a direct investigation into the relationship between running mechanics and RMS acceleration. The measurement of acceleration requires little equipment, can be done in the field, and real-time feedback is possible. Since the major movements of running are in the sagittal plane, we focused on the flexion/extension behavior of the hip, knee, and ankle joints during various gait events, as well as some other key variables that are readily modifiable. The purpose of this study was to determine the biomechanical factors contributing to global axial accelerations in active healthy males. In previous work [8], we observed greater accelerations in healthy untrained runners compared to trained collegiate runners. In the current study we selected a sample that was relatively heterogeneous with regard to chosen mode of physical activity and indicative of those from the general population who might take up running as a recreational activity for health benefits. These individuals would be more likely to exhibit mechanics that would make them more susceptible to injury due to relatively high accelerations [8]. To accomplish our objectives, we used a multiple regression approach to determine the variables that best fit a least squares model generated for RMS acceleration in each axis. Additionally, principal components analysis (PCA) was used to establish potentially hidden interactions between individual variables that can be combined to form separate components. These components may be then assessed for their contribution to axial accelerations. Thus, with a view to performance and injury management, this study will provide a description of modifiable biomechanical factors and their relationship with RMS trunk accelerations.

## Methods

### Subjects and experimental procedure

Eighteen healthy, active, college-age males volunteered to participate. Subjects participated 2–7 times per week in various forms of physical activity such as individual endurance sports (including running for 6 subjects), strength training, team sports, and/or combat sports. The procedures of this study were approved by the Human Subjects Review Committee of Eastern Michigan University College of Health and Human Services. All subjects provided written informed consent.

We analyzed 60 sec of data from three randomly-ordered treadmill run trials run at 2.22, 2.78, and 3.33 m∙s^−1^. Subjects were given as much rest between trials as they desired (typically 60–180 s). Subject characteristics are presented in Table 1.

**Table 1:** **Subject characteristics**

	Mean	SD	Min	Max
Age (yr)	24.0	4.2	19	32
Height (m)	1.78	0.07	1.66	1.89
Mass (kg)	79.7	14.8	59.1	107.3
BMI (kg∙m^−2^)	25.2	3.6	20.8	31.7

### Instrumentation

We placed one triaxial high resolution accelerometer (G-Link ADXL 210, Microstrain, Inc., Williston, VT) on the dorsal mid-line, at the level of the iliac crest (approximately at the L4/L5 spinous process). Accelerometers mounted at this anatomical location can provide valid estimates of oxygen consumption during running and can distinguish mechanics between trained and untrained individuals [8]. Although the legs primarily move in the sagittal plane during running, this is not the case for the spine and pelvis. Because the accelerometer is mounted in that region, there is significant non-sagittal movement requiring measurement in three and not just two dimensions. The accelerometer (mass = 47 g) consists of internal circuitry enclosed in a 58 × 43 × 21 mm casing, plus an antenna extending a bit outside the dimensions and adding 18 mm to the thickness. The accelerometer was mounted to a semi-rigid strap, and secured with elastic wrap to minimize extraneous movement of the device.

Kinematic data was collected with a 3-D optical motion capture system (Vicon MX, Vicon, Centennial, CO). We employed a 39-marker full body gait model (Plug-In-Gait, Vicon, Los Angeles, CA) consisting of 15 segments including the head, thorax, pelvis, upper arm, forearm, hand, thigh, shank, and foot. Seven cameras (Vicon T40 and T40 S) were placed roughly equidistant to the subject on the treadmill. Mean values for fourteen kinematic variables were calculated (mean value for left and right leg). Foot contact was defined as the point of lowest vertical displacement of the heel marker [19]. Mid-stance was defined as the lowest point of the software-estimated COM. Toe-off was defined as the point of maximum knee extension [19]. Lower limb joint angles were calculated according to the parameters of the software and model. Variables are listed and defined in Table 2.

**Table 2:** **Biomechanical variable definitions**

Abbreviation	Explanation	Measurement convention
Hip-max	Maximum hip angle (before foot-strike).	Positive = flexion
Hip-FS	Hip angle at foot-strike.	
Hip-MS	Hip angle at mid-stance.	
Hip-TO	Hip angle at toe-off.	
Knee-FS	Knee angle at foot-strike.	Positive = flexion
Knee-MS	Knee angle at mid-stance.	
Knee-TO	Knee angle at toe-off.	
Ankle-FS	Ankle angle at foot-strike.	Positive = dorsiflexion
Ankle-TO	Ankle angle at toe-off.	
PR	Mean range of pelvis rotation in the transverse plane for each gait cycle.	Scalar
FA	Foot advance; sagittal plane distance between the heel and COM at foot contact, relative to mean leg length.	Scalar
DROP	Vertical displacement of COM from foot contact to mid-stance, relative to mean leg length.	Scalar
RISE	Vertical displacement of COM from mid-stance to toe-off, relative to mean leg length.	Scalar
SR	Step rate.	Steps per min

### Data capture and analysis

Data were collected in the medio-lateral (ML), anterio-posterior (AP), and vertical (VT) axes. Trajectories were sampled at 200 Hz and then filtered with a 4th order Butterworth filter with a low pass cutoff at 10 Hz. Accelerometer data were streamed wirelessly at 617 Hz to Agilelink software (Microstrain, Williston, VT), subsequently re-sampled at 200 Hz, and filtered similarly to correspond with motion capture data. During running, the device is not perfectly aligned relative to the room (i.e., the global coordinate system, as opposed to the body coordinate system). Corrections were made for the tilt of the accelerometer, based on the method of Moe-Nilssen [9]. We provide a brief description of the calculations, but we encourage the reader to study the details provided in that paper [9]. Correction is possible because the mean vector angles in the ML and AP sensing axes may be estimated while the participant is running (see Appendix for calculations). The RMS of the vertical (VT_RMS_), medio-lateral (ML_RMS_), and anterior-posterior (AP_RMS_) axes was then calculated for the epochs in each trial:

1 $$x_{RMS}=\sqrt{\frac{1}{N}\sum_{i}^{N}{x_{i}}^{2}}$$

where *x* is the given plane and *N* is the total number of samples in 60 sec (at 200 Hz, *N* = 12,000). The resultant Euclidian scalar variable (RES) was also calculated for the determination of the magnitude of the overall body acceleration:

2 $$RES_{RMS}=\sqrt{V{T_{RMS}}^{2}+M{L_{RMS}}^{2}+A{P_{RMS}}^{2}}$$

The above processing and analysis of data was done using custom designed code in a Matlab environment (Matlab R2013b, Mathworks, Natick, MA).

### Statistical tests

Correlations were first performed to assess the relationship between anthropometric variables and acceleration. Analysis of variance (ANOVA) was used to determine the effect of speed on the four acceleration and fourteen biomechanical variables. A stepwise regression was then used to determine the significant kinematic contributions to acceleration in each axis. We also performed principal component analysis (PCA) to reduce the dimensionality of the data into significant components using a varimax rotation and Kaiser normalization. A stepwise regression was then performed using these components as predictors of acceleration in each axis. *Post hoc* power analyses were conducted for all ANOVA and regression analyses. A Bonferroni test was used for multiple comparisons, where appropriate. Statistical significance was set at p < 0.05. Statistical analysis was done using SPSS software (version 21, IBM Corporation, Armonk, NY).

## Results

Significant speed effects were found for RMS-accelerations for ML, AP, and RES (p < 0.05, Table 3). Of the biomechanical variables, only maximum hip angle showed a significant speed effect (p < 0.05, Table 3). Height, mass, and BMI were not significantly correlated with acceleration in any axis (p < 0.05).

**Table 3:** **Mean (SD) acceleration and biomechanical variables for each speed**

Variable	Speed (m/s)			Observed power
	2.22	2.78	3.33	
ML_RMS_ (g)*	0.35 (0.05)	0.41 (0.06)^†^	0.46 (0.07)^†^	1.00
AP_RMS_ (g)*	0.36 (0.06)	0.43 (0.10)^†^	0.50 (0.10)^†^	0.99
VT_RMS_ (g)	1.09 (0.13)	1.18 (0.11)	1.19 (0.10)	0.72
RES_RMS_ (g)*	1.21 (0.12)	1.33 (0.12)^†^	1.38 (0.11)^†^	0.99
Hip-max (deg)*	36.2 (6.8)	42.1 (7.2)^†^	48.3 (7.4)^†^	1.00
Hip-FS (deg)	28.9 (6.5)	31.5 (5.8)	34.6 (6.2)	0.68
Hip-MS (deg)	22.6 (7.6)	24.9 (6.9)	27.6 (7.4)	0.41
Hip-TO (deg)	−5.4 (5.6)	−8.4 (5.6)	−10.5 (5.6)	0.68
Knee-FS (deg)	13.0 (8.2)	12.3 (6.6)	13.7 (6.6)	0.08
Knee-MS (deg)	37.9 (7.1)	39.3 (6.8)	40.6 (6.7)	0.16
Knee-TO (deg)	10.5 (6.5)	8.6 (5.8)	8.0 (6.1)	0.19
Ankle-FS (deg)	9.4 (5.0)	8.9 (4.8)	9.2 (4.8)	0.06
Ankle-TO (deg)	−11.5 (7.5)	−16.0 (5.4)	−17.6 (5.7)	0.76
PR (deg)	3.9 (1.6)	4.7 (2.5)	5.6 (3.7)	0.34
FA (% mean leg length)	5.3 (2.9)	6.9 (3.0)	9.2 (3.8)	0.90
DROP (% mean leg length)	6.2 (1.7)	6.3 (1.5)	5.9 (1.4)	0.10
RISE (% mean leg length)	8.6 (1.7)	8.9 (1.5)	8.7 (1.5)	0.09
SR (steps/min)	155.5 (9.5)	158.0 (9.0)	163.1 (10.5)	0.54

*Significant speed effect at p < 0.05, ^†^significantly different from 2.22 m∙s^−1^.

**Figure 1:**
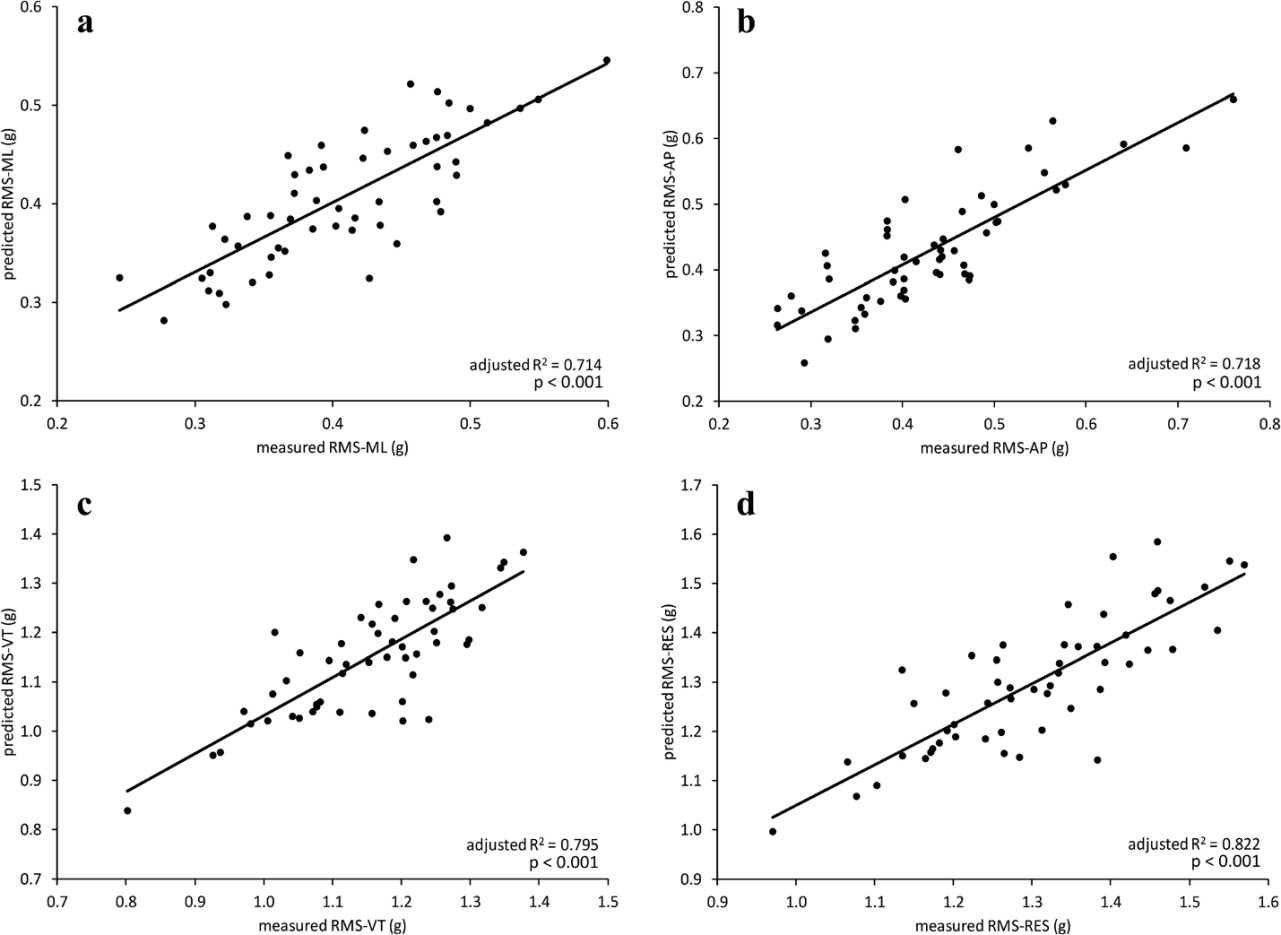
**Predicted RMS acceleration versus measured RMS acceleration values.** Graphs indicate: **(a)** ML, **b)** AP, **c)** VT, and **d)** RES. Each graph includes data from all three speeds. Most biomechanical variables did not show a speed effect.

Regression indicated 4 to 5 significant variables associated with acceleration, depending on the axis (Table 4). We encourage the reader to take notice of the sign of the beta coefficients (Table 4) and the angle definitions (Table 2) to understand the direction of change that is associated with an increase in acceleration. The combination of significant variables was different for each axis. Explained variance (R^2^) ranged from 0.71 to 0.82. A plot of predicted versus measured RMS acceleration for each axis is provided in Figure 1.

**Table 4:** **Regression results for original variables**

Dependent	Adjusted R^2^	Independent	Beta	*p*	Observed power
ML_RMS_	0.714^a^	Hip-max	0.009	< 0.001	1.00
		Hip-TO	−0.005	< 0.001	
		Knee-FS	−0.003	0.002	
		Knee-MS	−0.003	0.020	
AP_RMS_	0.718^b^	Hip-max	0.016	< 0.001	1.00
		Hip-MS	−0.010	< 0.001	
		Hip-TO	−0.008	< 0.001	
		FA	−0.010	< 0.001	
		RISE	−0.020	0.001	
VT_RMS_	0.795^c^	Hip-FS	0.034	< 0.001	1.00
		FA	−0.027	< 0.001	
		Hip-MS	−0.030	< 0.001	
		Hip-max	0.010	< 0.001	
		Ankle-TO	−0.014	< 0.001	
RES_RMS_	0.822^d^	Hip-Max	0.017	< 0.001	1.00
		FA	−0.028	< 0.001	
		Hip-FS	0.034	< 0.001	
		Hip-MS	−0.036	< 0.001	
		Ankle-TO	−0.014	< 0.001	

^a^F(4,51) = 35.362, p < 0.001; ^b^F(5,50) = 28.960, p < 0.001; ^c^F(5,50) = 45.542, p < 0.001. ^d^RES: F(5,50) = 51.967, p < 0.001.

PCA indicated 4 significant kinematic components (Table 5), explaining 79.1% of total variance. Component 1 (λ = 4.9, 37.4% of variance) was comprised of variables predominantly associated with hip flexion in late flight and early stance phase (hip-MS, hip-FS, knee-MS, hip-max). Component 2 (λ = 2.8, 21.2% of variance) was associated with the propulsive phase of the gait cycle (ankle-TO, knee-TO, RISE, PR). Component 3 (λ = 1.6, 12.5% of variance) included variables associated with cushioning during the early stance phase (knee-FS, DROP, ankle-FS). Regressions (Table 6) indicated that components 1 and 2 significantly predicted ML, VT, and RES acceleration (R^2^ from 0.32 to 0.40, p < 0.001). Component 3 significantly predicted AP acceleration (R^2^ = 0.041, p = 0.041).

**Table 5:** **Rotated component matrix from principal component analysis**

	Component			
	1	2	3	4
Hip-MS	0.910*	−0.058	0.081	−0.127
Hip-FS	0.906*	0.022	−0.016	0.134
Knee-MS	0.897*	0.034	0.239	0.213
Hip-max	0.852*	−0.146	0.157	0.105
Ankle-TO	−0.114	0.888*	−0.033	−0.295
Knee-TO	0.345	0.812*	0.076	0.069
RISE	0.599	−0.658*	−0.063	−0.009
PR	0.172	−0.517*	−0.054	−0.122
Knee-FS	0.342	−0.004	0.902*	−0.074
DROP	0.600	−0.006	−0.667*	0.243
Ankle-FS	0.505	0.305	0.514*	0.117
FA	0.352	0.062	−0.172	0.810*
Hip-TO	0.401	0.588	−0.112	−0.591*

Bold font and *indicates grouping for each component.

**Table 6:** **Regression results for principal components**

Dependent	Adjusted R^2^	Predictors	Beta	*p*	Observed power
ML_RMS_	0.322^a^	Component 1	0.418	< 0.001	1.00
		Component 2	−0.415	< 0.001	
AP_RMS_	0.058^b^	Component 3	0.273	0.041	1.00
VT_RMS_	0.401^c^	Component 2	−0.529	< 0.001	1.00
		Component 1	0.378	0.001	
RES_RMS_	0.380^d^	Component 2	−0.513	< 0.001	1.00
		Component 1	0.374	0.001	

^a^F(2,53) = 14.066, p < 0.001; ^b^F(1,54) = 4.364, p = 0.041; ^c^F(2,53) = 19.412, p < 0.001.

^d^F(2,53) = 17.875, p < 0.001.

## Discussion

The purpose of this study was to determine the biomechanical contributors to global axial RMS accelerations during running. We found significant relationships where explained variance using regressions on the original variables was 0.71 for ML, 0.53 for AP, 0.74 for VT, and 0.43 for RES. PCA did identify hidden relationships that explained 79% of the variance of the original variables and that were not evident using only multiple regression. When regressions were performed using the PCA component variables, though, explained variance was lower than with the original biomechanical variables alone. Reducing the numerous variables into a few principal components therefore does explain much of the variance in a simplified manner, but the predictive value of this simplified relationship is not as strong as using a traditional regression with a non-reduced variable set.

Accelerations measured at the lumbar spine originate from the GRF, which is transmitted through the foot, shank, thigh, and pelvis. GRF at the shank is typically biphasic and is significantly attenuated at proximal body segments [20, 21]. The two GRF peaks are associated with impact and propulsion [22, 23], with resultant body segment acceleration depending on GRF magnitude and damping effects [24]. The magnitude of force applied to the ground depends, in part, on the stiffness of the lower extremities, as does the acceleration resulting from the GRF.

According to the regressions, increased RMS accelerations were associated with different combinations of the following kinematic characteristics during early stance phase, depending on the axis: increased hip flexion, decreased knee flexion, and decreased foot advance. Most studies demonstrate that a combination of increased hip flexion and decreased ankle dorsiflexion at foot contact is associated with alterations in GRF during foot contact in various settings, providing evidence for the role of both the quadriceps and the ankle dorsiflexor muscles in shock absorption [25–30]. Our data did not demonstrate the importance of ankle dynamics in shock attenuation, but did highlight the role of hip angle in positioning the quadriceps for shock attenuation during running [21, 31]. Indeed, decreased FA was associated with increased AP, VT, and RES acceleration. In contrast, a greater FA leads to a flatter angle of attack (i.e., angle between segment and the ground), which results in high lengthening rates and decreased rate and magnitude of loading [32].

During the late stance and propulsion phase, a greater hip extension and ankle plantar flexion at toe off were associated with greater RMS acceleration. According to modeling by Hamner et al. [30], the soleus and gastrocnemius provide the biggest contributions to the propulsion phase. Data from the present study supports the important role of the ankle plantar flexors in propulsion.

Kinematic observations in the current study are similar to changes observed by McMahon et al. [16] when performing a “Groucho running” intervention. In that study, reductions in leg stiffness were associated with reduced GRF and increased metabolic cost and are accomplished by increased knee flexion. In contrast, in the present study this appears to be facilitated by increased hip joint excursion and a decreased foot advance. We note that Groucho running is an exaggerated style for the purpose of establishing a relationship, and not intended for exercise and performance purposes. The subjects in the present study used a freely-chosen technique and were not given any instruction to modify their form. Still, the kinematic descriptions we provide would seem to be subject to modification with skill training [17, 18].

There is also evidence that the level of acceleration may be modified with training. We have previously shown that the vertical accelerations of trained collegiate runners are lower than untrained individuals but greater than triathletes with similar fitness and training volume [33]. This may represent an optimization of the different performance requirements and injury risk between the different groups because the optimal magnitude of vertical accelerations for performance may be different than what is optimal for minimizing risk of injury, and both may be different from sport to sport. Acceleration magnitude and stiffness may reflect several aspects of physical function during running such as energy expenditure, impact forces relating to stress and injury, and performance [10, 11]. Often, one aspect must be compromised if another is to be maximized. For example, high impact forces accompanying high limb stiffness may increase energy return and performance according to the spring-mass model but may require more energy and occur at the expense of an overuse injury [32].

In the current study, we employ a simple approach to modeling, including kinematic descriptions and a single acceleration quantity for each axis (representing accelerations over the entire gait cycle); this work represents an easily accessible method with the potential for real-time output. Although it is not possible to fully account for the myriad of interactions between force, acceleration, stiffness, effective segmental mass, performance, and injury, there have been several reports of the benefits of interventions using acceleration as an outcome variable [17, 18, 34]. Data reduction via PCA facilitates the tracking of such characteristics because the number of features becomes relatively smaller [35]. Indeed, satisfactory descriptions of walking gait using PCA (~80-90% explained variance) applied to continuous waveforms of joint markers or joint ankles have been obtained with only the first three or four principal components [35–39]. However, sometimes only one of many principal components is significantly different between subject groups (fallers vs. non-fallers, overweight vs. normal weight) or experimental conditions (loaded vs. non-loaded) in walking tasks [40, 41]. The reduction of the kinematic variables into four principal components may aid conceptualization of the key gait characteristics that contribute to the magnitude of accelerations. That it was possible to form components from the different biomechanical variables is likely indicative of movement *synergies* employed by the individual as a motor strategy [37]. To the extent that this strategy can be altered, this presents an opportunity to modify force production and impact absorption.

The discrete values used in the present study represent an *a priori* reduction from continuous waveform data, and may be seen as a limitation, but the maximum and minimum values found in a waveform can often be the regions of most significant difference [40], and would thus likely be captured at points during the gait cycle that we examined. As well, the complete dataset of biomechanical variables displayed greater explained variance than the principal components. This may indicate that the reduction of the complete dataset results in the loss of important information that is explanatory with regard to gait dynamics. However, this does not necessarily diminish the value in identifying otherwise hidden synergistic relationships perhaps indicative of a neuromuscular strategy. Another limitation is the small number of biomechanical variables chosen for analysis. While the selection of a few readily modifiable variables provides a simple preliminary analysis, there are other variables that have not been included that potentially affect RMS trunk acceleration. Indeed, our measurements focused on movement in the sagittal plane, but this neglects frontal plane dynamics that may influence medio-lateral acceleration. Because the accelerometer only approximates COM movement, the findings are limited if an explanation of COM *per se* is desired. However, if the goal is to investigate what contributes to measured accelerations, and explain previous findings (c.f. McGregor [8]) then the factors highlighted in this paper provide a basis for future investigations.

## Conclusions

This study helps to establish the use of lumbar-mounted accelerometers to demonstrate effects related to stiffness, impact, and the attenuation of acceleration. Previous work has demonstrated the connection between RMS accelerations and energy expenditure [8]. Our present data provides a more mechanistic explanation of how various kinematic configurations may influence the multi-segmental force cascade from the foot-ground interface to the lumbar vertebrae where accelerations are measured. Specifically, we have identified the role of hip and knee angles in shock absorption and the role of the hip and ankle in propulsion. In addition to establishing these key biomechanical contributors to acceleration, we showed how many of these variables change in concert. Wherever these variables are modifiable, the acceleration signal may be a useful way to monitor movement with a view to performance and injury management. Our findings pertain to young, healthy, and active men and women, but the relationships found here can form the basis from which more specific subject groups may be studied in the future.

## Appendix

### Statistical tests

The following steps and equations are based on Moe-Nilssen [9]. For a dataset of large N, the acceleration vector approaches the sine of the angle of that vector:

3 $$\lim{\bar{a}}_{\mathrm{ML}}=\sin\theta_{\mathrm{ML}}$$

4 $$\lim{\bar{a}}_{AP}=\sin\theta_{AP}$$

The coordinate system definitions must be strictly maintained so that the positive/negative signs are correct and the relationships hold true. The following corrections were applied to each of the axes:

5 $$a_{\mathrm{APcorr}}=a_{\mathrm{APmeas}}\cdot \cos\theta_{AP}-a_{\mathrm{VTmeas}}\cdot \sin\theta_{AP}$$

6 $$a_{\mathrm{VTprov}}=a_{\mathrm{APmeas}}\cdot \sin\theta_{AP}+a_{\mathrm{VTmeas}}\cdot \cos\theta_{AP}$$

7 $$a_{\mathrm{MLcorr}}=a_{\mathrm{MLmeas}}\cdot \cos\theta_{\mathrm{ML}}-a_{\mathrm{VTprov}}\cdot \sin\theta_{\mathrm{ML}}$$

8 $$a_{\mathrm{VTcorr}}=a_{\mathrm{MLmeas}}\cdot \sin\theta_{\mathrm{ML}}+a_{\mathrm{VTprov}}\cdot \cos\theta_{\mathrm{ML}}+1$$

where *corr*, *meas*, and *prov* refer to corrected, measured, and provisional terms, respectively. The static component of gravity was also corrected for the VT axis, leaving only the dynamic acceleration component.

## Authors’ original submitted files for images

Below are the links to the authors’ original submitted files for images. Authors’ original file for figure 1
