# Supplementary figures and images for: Contributions of lower extremity kinematics to trunk accelerations during moderate treadmill running
Source: J Neuroeng Rehabil. 2014 Dec 12;11:162. doi: 10.1186/1743-0003-11-162 (PMC4326429; doi:10.1186/1743-0003-11-162)

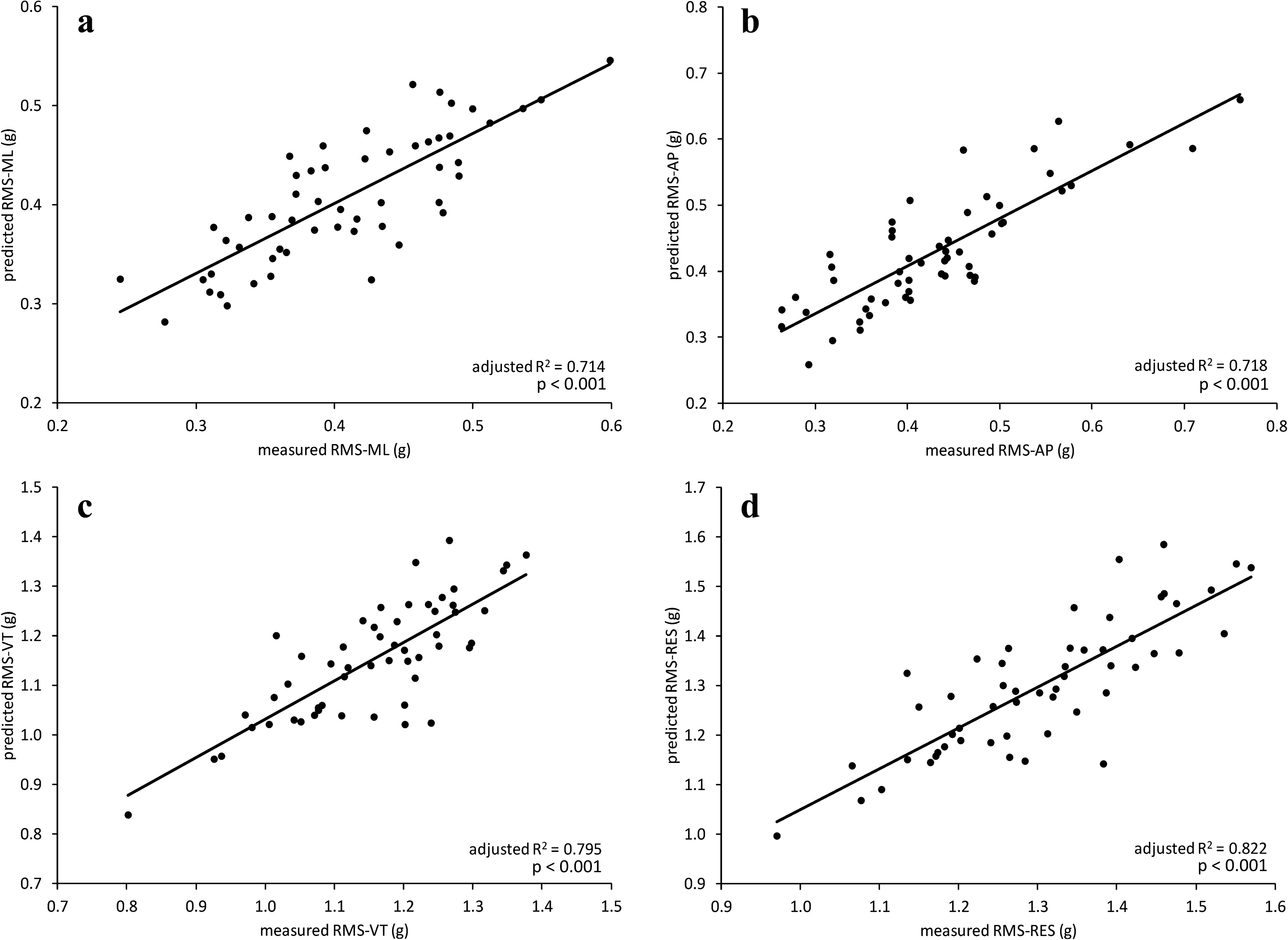

Supplement: Supplementary file 1 — Authors’ original file for figure 1 [file 12984_2013_701_MOESM1_ESM.tif]
